# Hypoargininemia exacerbates airway hyperresponsiveness in a mouse model of asthma

**DOI:** 10.1186/s12931-018-0809-9

**Published:** 2018-05-23

**Authors:** Roy H. E. Cloots, Matthew E. Poynter, Els Terwindt, Wouter H. Lamers, S. Eleonore Köhler

**Affiliations:** 1 Department of Anatomy & Embryology and NUTRIM School of Nutrition and Translational Research in Metabolism, Maastricht University, P.O. Box 616, 6200MD, Maastricht, The Netherlands; 2 Tytgat Institute for Liver and Intestinal Research, Academic Medical Center, Amsterdam, The Netherlands; 3 Department of Medicine, College of Medicine, Division of Pulmonary Disease and Critical Care, University of Vermont, VT, Burlington, USA

**Keywords:** Arginine availability, Lung function, Transgenes, Pulmonary inflammation

## Abstract

**Background:**

Asthma is a chronic respiratory condition, with airway hyperresponsiveness (AHR) and inflammation as hallmarks. The hypothesis that the substantially increased expression of arginase 1 in activated macrophages limits the availability of L-arginine for nitric oxide synthesis, and thus increases AHR in lungs of mice with experimentally induced allergic asthma was recently refuted by several studies. In the present study, we tested the hypothesis that, instead, a low circulating concentration of arginine aggravates AHR in the same murine asthma model. Female FVB *F/A2*^*tg/tg*^ transgenic mice, which overexpress rat arginase 1 in their enterocytes, exhibit a ~ 50% decrease of their plasma L-arginine concentration.

**Methods:**

Adult female *F/A2*^*tg/tg*^ mice and their wild-type littermates (*F/A2*^*wt/wt*^) were sensitized and challenged with ovalbumin (OVA/OVA). Lung function was assessed with the flexiVent™ system. Adaptive changes in the expression of arginine-metabolizing or -transporting enzymes, chemokines and cytokines, and lung histology were quantified with qPCR, ELISA, and immunohistochemistry, respectively.

**Results:**

Reduction of circulating L-arginine concentration significantly increased AHR in OVA/OVA-treated mice and, to a lesser extent, even in PBS/OVA-treated mice. The pulmonary inflammatory response in OVA/OVA-treated *F/A2*^*tg/tg*^ and *F/A2*^*wt/wt*^ mice was comparable. OVA/OVA-treated *F/A2*^*tg/tg*^ mice differed from similarly treated female mice, in which arginase 1 expression in lung macrophages was eliminated, by a complete absence of an adaptive increase in the expression of arginine-metabolizing or –transporting enzymes.

**Conclusion:**

A reduction of the circulating L-arginine concentration rather than the macrophage-mediated increase of arginine catabolism worsens AHR.

**Electronic supplementary material:**

The online version of this article (10.1186/s12931-018-0809-9) contains supplementary material, which is available to authorized users.

## Background

Allergic asthma is a chronic respiratory condition characterized by T_H_2-predominant airway inflammation, increased mucus production, mucosal edema, airway hyperresponsiveness (AHR) and airway remodeling. Expression of the enzyme arginase is increased in asthmatic lungs. It has been suggested that increased arginase expression affects AHR by reducing L-arginine availability for nitric oxide synthases (NOS), thereby limiting the production of nitric oxide (NO) [1, 2]. However, the relation appears to be complex. The contribution of the individual NOS enzymes to bronchodilation or AHR is difficult to determine since all three NOS isoforms are expressed in the lung and control each other’s expression and activity [3]. Furthermore, L-arginine availability is not directly related to exhaled NO or inflammatory parameters in asthma, and is related to AHR only in severe asthma [4]. Intracellular L-arginine availability depends on its consumption by NO synthases and arginases, and its uptake into the cell through transporters, such as cationic (CATs) and heterodimeric amino-acid transporters (e.g. LAT1 and − 2), and on recycling of L-citrulline (the other product of NOS activity) to L-arginine. CAT-mediated uptake of arginine is inhibited by other cationic amino acids, such as L-ornithine (a product of arginase activity) and lysine, but also by polycations, such as major basic protein (MBP; [5]), which is released by infiltrating eosinophils during the asthmatic response.

The effects of elimination of NOS isoforms [6–10] or arginase 1 [11, 12] on lung function and inflammation in mouse models of allergic asthma have been extensively investigated. In these animal models, NOS2 [9], arginase 1 [11, 13] and, to a lesser extent, arginase 2 [14] are strongly upregulated. The more predominant arginase form, arginase 1, is found in macrophages within the inflammatory infiltrates in the asthmatic lung [13]. The potential competition of arginase and NOS for their common substrate arginine explains the interest in the behavior of both enzymes. Although NO synthases have a ~ 250-fold higher affinity for L-arginine than arginases, arginases have a ~ 1000-fold higher V_max_ than the NO synthases, which makes regional depletion of arginine [15] and uncoupling of NOS [16] a realistic possibility.

Although arginase 1 elimination in macrophages had major effects on the gene-expression profile of pulmonary macrophages, the effects on lung function were absent in both BALB/c and C57BL/6 mice [12, 17, 18], or limited to the peripheral airways in male C57BL/6 mice [11]. We, therefore, hypothesized that macrophages were less important for the regulation of local arginine availability in the airways than anticipated and that, instead, the circulating concentration of arginine played the more important role in supplying arginine for NO synthesis and relaxation of airway smooth muscles. To test this hypothesis, we studied mice in which the circulating L-arginine concentration was decreased to ~ 45% of control mice due to overexpression of arginase 1 in the enterocytes of their intestines [19] and sensitized and exposed them to ovalbumin to induce an asthmatic phenotype.

## Methods

### Animals

*F/A2* transgenic mice on a FVB background, which overexpress rat arginase 1 in their enterocytes [19], were bred hemizygously (*F/A2*^*wt/tg*^). We studied female homozygous (*F/A2*^*tg/tg*^) mice and used their female wild-type (*F/A2*^*wt/wt*^) littermates as controls. All mice were approximately 10 weeks at the start of the experiments. The study was reviewed and approved by the committee for animal care and use of Maastricht University (DEC2005–146).

### Antigen sensitization and challenge

Mice were injected intraperitoneally on days 0 and 14 with 10 μg ovalbumin (OVA), grade V (Sigma-Aldrich, Zwijndrecht, The Netherlands) in a total volume of 100 μL PBS, containing 1 mg/mL of AlOH_3_ (alum) as adjuvant (Imject Alum®, Thermo Scientific, Rockford, IL, USA). To ensure an adequate induction of gene expression in the sensitized and challenged mice, we performed a time-course study. Additional file 1: Figure S1 shows that daily exposure to 1% OVA for 30 min strongly increased *Arg1*, *Nos2*, and *Cd68* expression after 6 days of treatment. Mice were, therefore, exposed on days 21–27 to daily aerosol challenges with 1% (*w*/*v*) OVA in PBS for 30 min in custom-made inhalation chambers. Lung function was assessed 12 h after the last challenge.

### Airway hyperresponsiveness

The protocol was described previously [11]. In brief, female mice were injected intraperitoneally with 80 mg/kg sodium pentobarbital to induce anesthesia, followed by 40 mg/kg after 30 min for anesthesia maintenance. An 18-gauge blunt needle was inserted into the trachea and connected to a mechanical small-animal ventilator (flexiVent™, Scireq, Montreal, Canada). The mice were ventilated at 200 breaths/min with a delivered tidal volume of 0.25 mL against a positive end expiratory pressure (PEEP) of 3 cm H_2_O applied by a water trap. Aerosolized methacholine (Sigma, Steinheim, Germany) challenges were performed by delivering successively 0, 3.1, 12.5 and 50.0 mg/mL methacholine in PBS. Following each aerosol challenge, ventilation was interrupted every 10 s to allow for a 1-s passive expiration followed by a 2-s broad-band (1–19.6 Hz) volume perturbation. The peak-to-peak excursion of the ventilator piston during delivery of these perturbations was 0.17 mL, resulting in a delivery of ~ 0.14 mL after correcting for gas compression in the ventilator cylinder and connecting tubing. Pressure and flow were recorded during application of the perturbations and used to calculate the input impedance (Z_rs_) of the respiratory system. Z_rs_ was then fitted to the uniformly ventilated model of the lung with constant-phase tissue impedance [20, 21].

### Plasma collection and analysis

On day 27, after completing the Flexivent analysis, blood was collected from the inferior caval vein in heparin-coated tubes, centrifuged for 3 min at 5000*g, snap frozen in liquid nitrogen and stored at − 80 °C. Plasma OVA-specific IgE levels were determined by ELISA (Product #: M036005, MD Biosciences, Zürich, Switzerland). For the determination of plasma amino acids, 50 μL of plasma was added to 4 mg sulfosalicylic acid, vortexed, snap-frozen in liquid nitrogen and stored at − 80 °C until use. Plasma amino acid concentrations were measured using a fully automated HPLC system [22].

### Tissue isolation

Immediately following euthanasia, lungs were isolated. The left lung was filled with 4% formaldehyde (Klinipath, Deventer, the Netherlands) for 10 min at a pressure of 20 cm H_2_O and submersed for 24 h in 4% formaldehyde at room temperature (RT) prior to paraffin embedding. The right lung was snap-frozen in liquid nitrogen and pulverized in a liquid-nitrogen-chilled mortar and pestle. Lung tissue powder was stored at -80 °C until further use.

### Immunostaining

Paraffin-embedded tissue was cut into 4 μm sections and stained with haematoxylin & eosin. For immunostaining, antigens were retrieved by heating the slides for 5 min in 10 mmol/L sodium citrate (pH 6) at 95 °C and cooling to room temperature (RT) in 30 min before blocking endogenous peroxidases with peroxidase block (DAKO, S2001, Enschede, the Netherlands) for 10 min at RT. This step was omitted if antibody binding was visualized with the alkaline phosphatase (AP) system. Sections were blocked with 10% normal goat serum for 30 min, followed by incubation with anti-arginase 1(Amsterdam Liver Center, AMS40.11.13), anti-myeloperoxidase (MPO; DAKO), or anti-murine major basic protein (mMBP; LeeLab, MT14.3.7, Mayo Clinic Scottsdale, AZ, USA) [23]. After washing, sections were incubated with a 1:200 diluted biotinylated rabbit anti-rat secondary antibody (DAKO) for 45 min at RT. Sections were washed, incubated with streptavidin/HRP (Vector) for 30 min at room temperature, and developed with 3,3′-diaminobenzidine (Sigma, Steinheim, Germany) for 10 min. Sections stained for arginase 1 were incubated with an AP-labeled secondary antibody (DAKO) for 45 min, developed with NitroBlue-Tetrazolium and 5-Bromo-4-Chloro-3-Indolyl phosphate (Roche, Almere, The Netherlands) dissolved in 50 mmol/L MgSO_4_, 100 mmol/L Tris·HCl (pH 9.5) for 30 min, and cover-slipped with an aqueous mounting medium (DAKO).

### Histopathology

Whole-lung tissue sections stained with H&E, or the MBP and MPO antisera were scored independently by two persons at 10X magnification. Scores were: 0: < 5 inflammatory cells per field; 1: 5–25 inflammatory cells per field; 2: 25–50 inflammatory cells per field; 3: > 50 inflammatory cells per field.

### Milliplex assay

Lung tissue powder was homogenized in PBS, pH 7.6, in the presence of a proteinase inhibitor cocktail (Roche, Complete). Cytokines (IL-4, IL-10, and IL-13) were quantified using a Luminex® xMAP® multiplex platform, combined with a customized Milliplex™ mouse chemokine/cytokine panel (Merck, Amsterdam, the Netherlands).

### RNA isolation and quantification

Lung tissue powder was homogenized in Tri reagent (Sigma) with the Mini Bead-Beater (Biospec products, Bartlesville, OK, USA). To remove genomic DNA, RNA was precipitated with 2 mol/L LiCl for at least 30 min at -20 °C. RNA integrity was checked by denaturing gel electrophoresis. RNA concentration was determined with a NanoDrop-ND-1000 spectrophotometer at 260 nm (Isogen Life Sciences, Wilmington, DE, USA). 400 ng of total RNA was transcribed using the Roche first-strand synthesis kit (Roche). Quantitative PCR was performed in the Lightcycler 480 (Roche), using the Lightcycler 480® SYBRgreen mastermix (Roche) and the following settings: denaturation 30 s at 95 °C; annealing 30 s at 60 °C; elongation 30 s at 72 °C; 45 cycles; and a final elongation step for 5 min at 72 °C. If reverse transcriptase was omitted, no product was formed. Primary fluorescent data were exported and analyzed with the Lin-Reg Analysis program [24]. mRNA abundance was expressed relative to *18S* rRNA abundance. Primer sequences are given in Additional file 2: Table S1.

### Western blot

Lung tissue powder was homogenized in RIPA buffer: 25 mmol/L Tris·HCl,pH 7.6, 150 mmol/L NaCl, 1% NP-40, 1% Na-deoxycholate, 0.1% SDS, containing Complete® cocktail (Roche). Protein concentration was measured with the bicinchoninic-acid assay (Pierce, Rockford, IL, USA). 25 μg protein was separated on an SDS-polyacrylamide gel, transferred onto 0.45 μm nitrocellulose membranes, using a wet transfer system (Biorad, Hercules, CA, USA), stained with Ponceau S to confirm equal loading of lanes, washed with TBS (50 mmol/L Tris, 150 mmol/L NaCl, pH 7.6) and blocked with 5% skimmed milk in TBS/ 0.5% Tween-2. Arginase 1 was visualized with rabbit anti-arginase 1 antibody (1:200), followed by an HRP-conjugated swine anti-rabbit secondary antibody (DAKO). The signal was developed using the Super Signal West Pico Substrate (Pierce) and quantified with the Fuji systems darkbox (Fuji Film Life Sciences, Tokyo, Japan).

### Statistical analyses

Comparison of groups was performed using the Kruskal-Wallis test for PBS/OVA- versus OVA/OVA-treated, and *F/A2*^*wt/wt*^ versus *F/A2*^*tg/tg*^ mice. Only when this nonparametric test indicated a difference between experimental groups, a multiple comparison of the groups was carried out. Values were considered statistically significant if *P* < 0.05, and as indicating a trend if *P* < 0.1.

The bivariate, two-tailed Spearman correlation coefficients between each of the lung-function parameters, mRNA and protein concentrations, histology scores and plasma amino-acid concentrations were determined after combining the data from the comparable PBS/OVA and OVA/OVA groups. In the Tables, *P*-values of the resulting correlation coefficients were color-coded, with red indicating *P* < 0.001, orange 0.01 > *P* > 0.001, and yellow 0.05 > *P* > 0.01.

## Results

### Hypoargininemia does not affect the response to allergic asthma

**Fig. 1:**
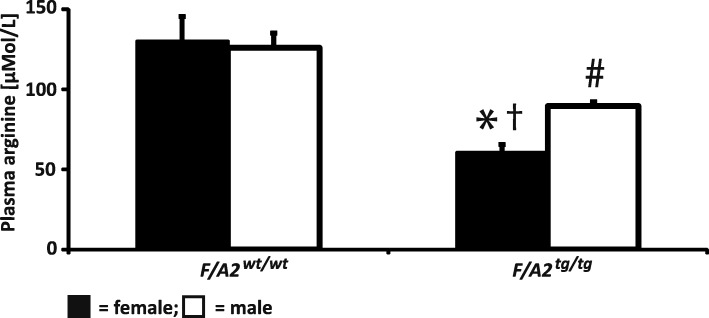
Decline of plasma arginine concentrations in female and male *F/A2*^*tg/tg*^ mice. Black bars represent female and white bars male mice. Means ± SEM of 7–8 mice per group. Significance symbols: * = *P* < 0.01 *F/A2*^*tg/tg*^ mice vs. *F/A2*^*wt/wt*^ (females); # = *P* < 0.01 *F/A2*^*tg/tg*^ mice vs. *F/A2*^*wt/wt*^ (males); † = *P* < 0.05 female vs. male *F/A2*^*tg/tg*^ mice

**Fig. 2:**
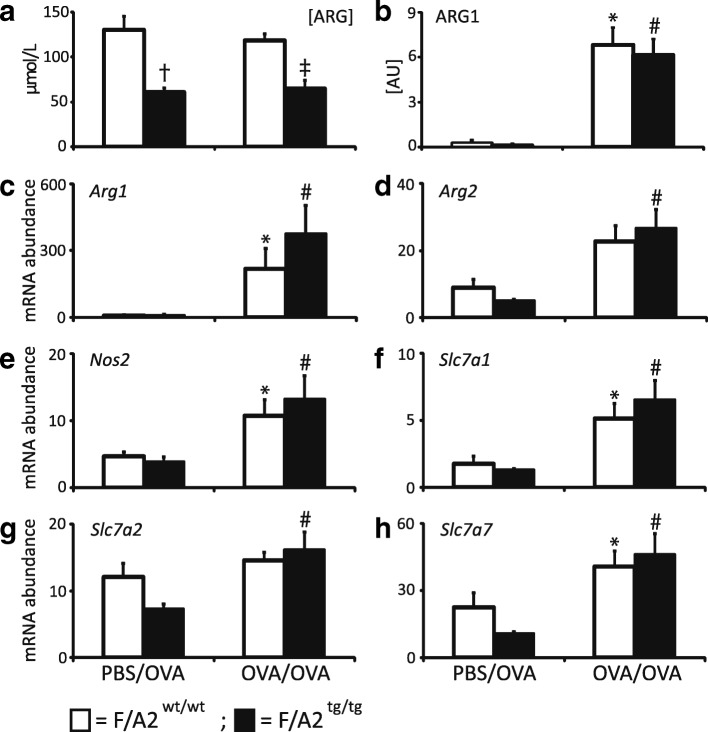
Similar induction of arginine-metabolizing enzymes and arginine transporters in asthmatic hypoargininemic and control mice. Mice were subjected to either the PBS/OVA (control) or the OVA/OVA treatment protocol as indicated at the bottom. Panel **a**: plasma arginine concentration; Panel **b**: arginase 1 protein concentration in lung tissue as determined by Western blot. Panels **c**-**e**: mRNA abundance of the arginine-metabolizing enzymes *Arg1, Arg2,* and *Nos2*, respectively. Panels **f**-**h**: mRNA abundance of the arginine transporters *Slc7a1, Slc7a2* and *Slc7a7*. White bars represent data from *F/A2*^*wt/wt*^ and black bars from *F/A2*^*tg/tg*^ *mice.* The Y-axis shows the number of mRNA copies after normalization to 18S rRNA expression and multiplication by 10,000. Means ± SEM of 9–10 mice per group. * = *P* < 0.05 PBS/OVA vs. OVA/OVA *F/A2*^*wt/wt*^; † = *P* < 0.05 PBS/OVA *F/A2*^*tg/tg*^ vs. *F/A2*^*wt/wt*^; # = *P* < 0.05 PBS/OVA vs. OVA/OVA *F/A2*^*tg/tg*^; ‡: *P* < 0.05 OVA/OVA *F/A2*^*tg/tg*^ vs. *F/A2*^*wt/wt*^

Overexpression of arginase 1 in the enterocytes of the small intestine decreases plasma arginine concentration, in particular in female mice (Figs. 1 and 2). In male *F/A2*^*tg/tg*^ mice, plasma arginine concentration was ~ 90 μmol/mL and in female *F/A2*^*tg/tg*^ mice ~ 63 μmol/mL, or ~ 70% and ~ 50% of their wild-type male and female littermates, respectively (Fig. 1). Because of their more pronounced hypoargininemia we confined our study to female mice. Treatment of female control (*F/A2*^*wt/wt*^*)* and transgenic (*F/A2*^*tg/tg*^*)* mice with the PBS/OVA or the OVA/OVA protocol did not affect plasma arginine concentration significantly (Fig. 2a). Since plasma concentrations of all other amino acids did not change between conditions or genotypes (Additional file 2: Table S2), arginine bioavailability indices such as [Arg]/([Orn] + [Lys]) (competition for CAT transporters) and [Arg]/([Orn] + [Cit]) (intracellular metabolism of arginine) declined correspondingly. Induction of experimental asthma increased arginase 1 expression in lungs dramatically (Fig. 2b), as observed previously [11–13]. We next investigated whether hypoargininemia was associated with adaptive changes in the expression of arginine-metabolizing enzymes or arginine transporters in the lung under allergically inflamed conditions. The pulmonary mRNA abundance of the arginine-metabolizing enzymes *Arg1, Arg2, Nos2* (Fig. 2c-e)*,* and the arginine transporters *Slc7a1, Slc7a2* and *Slc7a7* (Fig. 2f-h) did not differ significantly between *F/A2*^*tg/tg*^ and *F/A2*^*wt/wt*^ mice under control conditions (PBS/OVA protocol) All mRNAs increased in both *F/A2*^*wt/wt*^ and *F/A2*^*tg/tg*^ mice when exposed to the OVA/OVA protocol (Fig. 2), but the increase did not reach significance for *Arg2* and *Slc7a2* in *F/A2*^*wt/wt*^ mice.

### Hypoargininemia exacerbates allergen-induced airway hyperresponsiveness

**Fig. 3:**
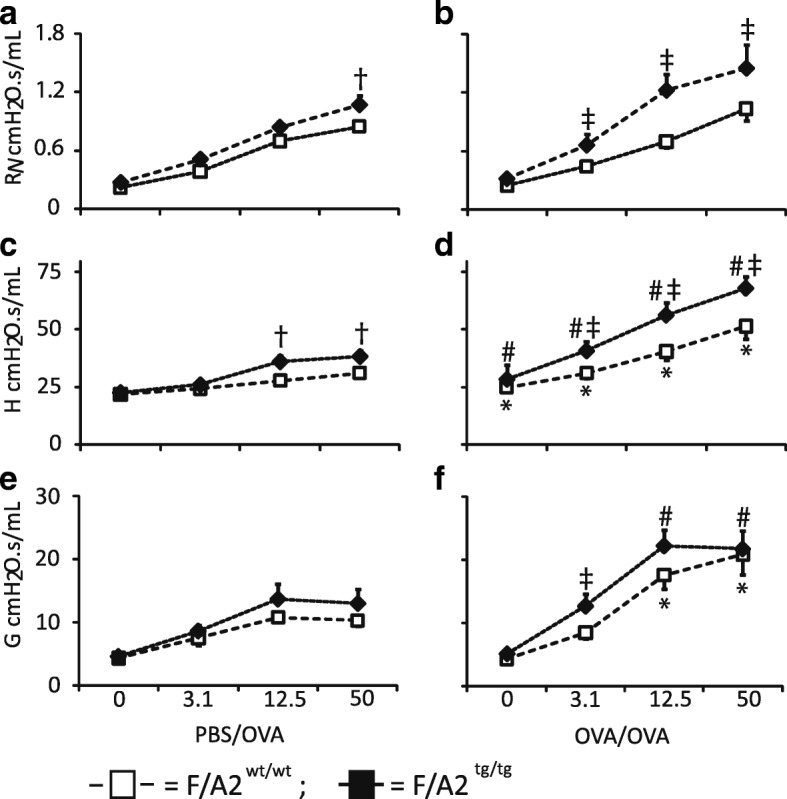
Hypoargininemia aggravates lung dysfunction in asthmatic mice. Panels **a**, **c**, and E show data from *F/A2*^*wt/wt*^ (white squares, continuous lines) and *F/A2*^*tg/tg*^ mice (black diamonds, dashed lines), mock-sensitized with PBS and challenged with ovalbumin (OVA). Panels **b**, **d**, and **f** show data from *F/A2*^*wt/wt*^ and *F/A2*^*tg/tg*^ mice that were sensitized and challenged with OVA. The treatment of the mice (PBS/OVA or OVA/OVA) is indicated below the columns. Top row: airway resistance R_N_; middle row: tissue elastance H; bottom row: tissue resistance G. Values on the X-axis represent the methacholine concentration used [mg/mL]. Means ± SEM of 9–10 mice per group. Significance symbols: *: *P* < 0.05 PBS/OVA vs. OVA/OVA *F/A2*^*wt/wt*^; †: *P* < 0.05 PBS/OVA *F/A2*^*tg/tg*^ vs. *F/A2*^*wt/wt*^; #: *P* < 0.05 PBS/OVA vs. OVA/OVA *F/A2*^*tg/tg*^; ‡: *P* < 0.05 OVA/OVA *F/A2*^*tg/tg*^ vs. *F/A2*^*wt/wt*^

To answer the question whether hypoargininemia has an effect on respiratory mechanics, we measured lung function in PBS/OVA- and OVA/OVA-treated *F/A2*^*tg/tg*^ and *F/A2*^*wt/wt*^ mice. Airway resistance (R_N_) in response to methacholine-challenges did not differ between PBS/OVA and OVA/OVA-treated wild-type mice (Fig. 3a, b), but was significantly higher in *F/A2*^*tg/tg*^ mice undergoing OVA/OVA treatment (Fig. 3b). Interestingly, a significantly higher R_N_) was already detected in PBS/OVA-treated *F/A2*^*tg/tg*^ mice challenged with 50 mg/mL of aerosolized methacholine when compared to PBS/OVA-treated *F/A2*^*wt/wt*^ mice (Fig. 3a). Both wild-type and transgenic mice showed significantly increased tissue elastance (H) (Fig. 3c, d) and resistance (G; Fig. 3e, f) when treated with the OVA/OVA protocol and challenged with methacholine. The increase of tissue elastance in response to methacholine was significantly higher in transgenic compared to wild-type mice (Fig. 3c, d), with the transgenic mice already showing an increased response to higher concentrations of methacholine on the PBS/OVA protocol. Transgenic mice were more sensitive to methacholine in terms of tissue resistance (*P* = 0.0004 at 3.1 mg/mL methacholine; *P* = 0.0001 at 12.5 mg/ml methacholine) under the OVA/OVA protocol. Tissue resistance did not differ between genotypes under control conditions (PBS/OVA). In aggregate, these data show that hypoargininemia increased all aspects of airway responsiveness measured with the flexiVent™ in mice with allergic asthma.

### Hypoargininemia does not affect induction of inflammatory genes in allergically inflamed lungs

**Fig. 4:**
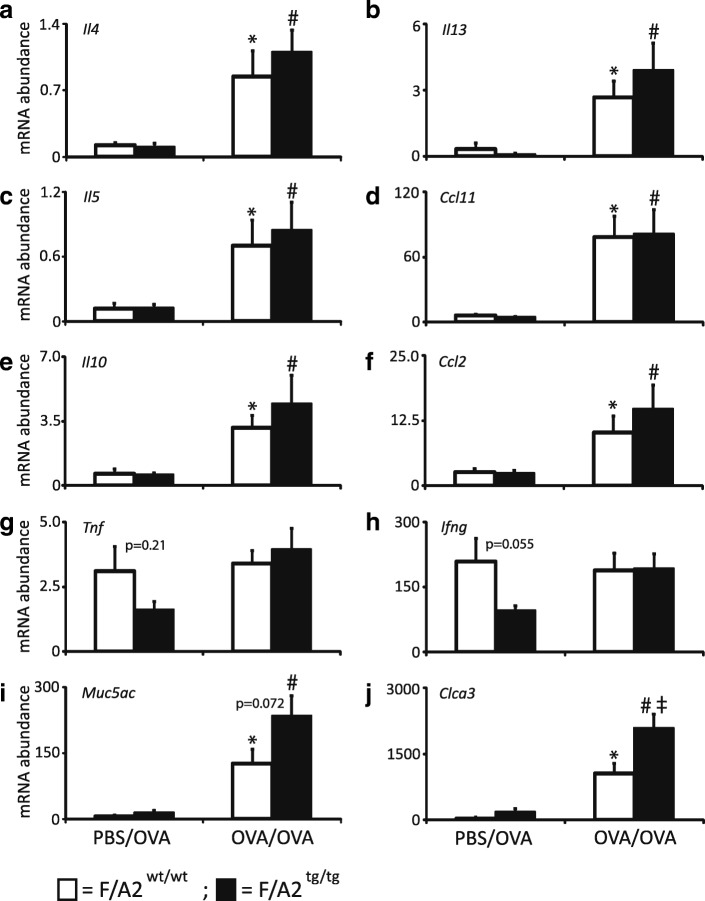
Similar induction of inflammatory genes in asthmatic hypoargininemic and control mice. Data from *F/A2*^*wt/wt*^ (white bars) and *F/A2*^*tg/tg*^ *(*black bars) mice are shown. Mice were either mock-sensitized with PBS before the ovalbumin (OVA) challenge (PBS/OVA), or sensitized and challenged with OVA (OVA/OVA) as indicated below panels I and J. Panels **a**-**d**: mRNA abundance of the T_H_2-related genes *Il4, Il13, Il5* and *Ccl11, respectively*. Panels **e** and **f**: mRNA abundance of anti-inflammatory gene *Il10* and macrophage-chemotactic gene *Ccl2*. Panels **g** and **h**: mRNA abundance of the T_H_1-related genes *Tnfa* and *Ifng*. Panels **i** and **j**: mRNA abundance of marker genes for activation of bronchiolar epithelium *Muc5ac* and *Clca3*. The Y-axis shows the number of mRNA copies after normalization to 18S rRNA expression and multiplication by 10,000. Means ± SEM of 7–8 mice per group. Significance symbols: *: *P* < 0.05 PBS/OVA vs. OVA/OVA *F/A2*^*wt/wt*^; #: *P* < 0.05 PBS/OVA vs. OVA/OVA *F/A2*^*tg/tg*^; ‡: *P* < 0.05 OVA/OVA *F/A2*^*tg/tg*^ vs. *F/A2*^*wt/wt*^

We next investigated whether hypoargininemia affected the gene expression of asthma-associated cytokines (Fig. 4). Allergen sensitization and challenge (OVA/OVA) resulted in an increase in the abundance of *Il4, Il5, Il13, Ccl2, Ccl11,* and *Il10* mRNAs in both *F/A2*^*tg/tg*^ and *F/A2*^*wt/wt*^ mice. Furthermore, the expression of the respiratory epithelium-specific *Muc5ac* and *Clca3* genes was significantly increased, whereas the expression of *Tnfa* and *Ifng* remained unchanged. *Clca3* mRNA expression was significantly increased in OVA/OVA-treated *F/A2*^*tg/tg*^ compared to their wild-type counter parts.

### Hypoargininemia does not affect pulmonary levels of cytokines in allergically inflamed lungs

**Fig. 5:**
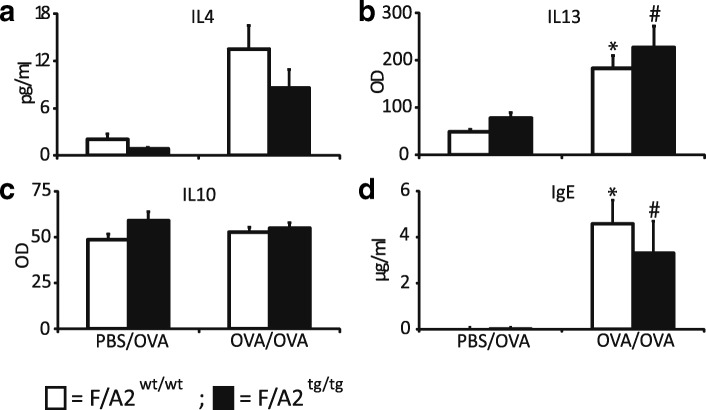
Similar changes in pulmonary content of inflammatory cytokines and OVA-specific IgE in asthmatic hypoargininemic and control mice. *F/A2*^*wt/wt*^ (white bars) and *F/A2*^*tg/tg*^ mice *(*black bars) were either mock-sensitized with PBS and challenged with ovalbumin (OVA; PBS/OVA), or sensitized and challenged with OVA (OVA/OVA) as indicated below the graphs. Cytokine or IgE concentrations were determined in lung tissue powder. Panel **a**-**d**: concentrations of IL4, IL13, IL10, and IgE, respectively, in plasma. The concentrations of IL13 and IL10 are expressed in arbitrary units. Means ± SEM of 7–8 mice per group. Significance symbols: *: *P* < 0.05 PBS/OVA vs. OVA/OVA *F/A2*^*wt/wt*^; #: *P* < 0.05 PBS/OVA vs. OVA/OVA *F/A2*^*tg/tg*^

To investigate whether hypoargininemia had an effect on the protein concentration of pulmonary cytokines that are involved in allergic airway inflammation, we performed ELISAs for IL-4, IL-10, and IL-13 in protein extracts of whole-lung homogenates (Fig. 5). Both IL-4 (trend *p* = 0.057) and IL-13 were induced by the OVA/OVA treatment to a similar extent in *F/A2*^*tg/tg*^ and *F/A2*^*wt/wt*^ mice, whereas IL-10 was not affected by the OVA/OVA treatment in both groups. OVA-specific IgE production was increased to a similar extent upon OVA/OVA treatment in both *F/A2*^*tg/tg*^ and *F/A2*^*wt/wt*^ mice.

### Hypoargininemia does not affect tissue inflammation in allergically inflamed lungs

**Fig. 6:**
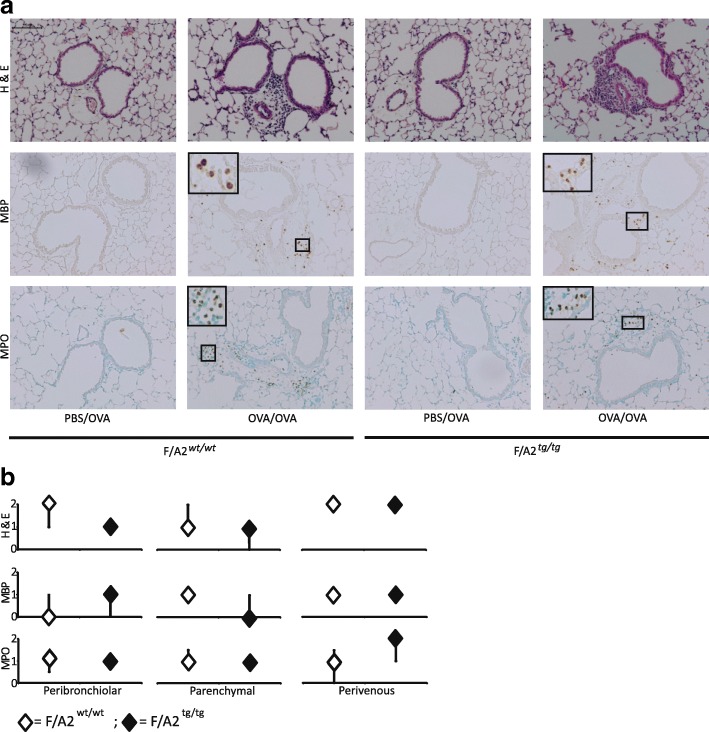
Similar prevalence of inflammatory cells in the lungs of asthmatic hypoargininemic and control mice. *F/A2*^*wt/wt*^ and *F/A2*^*tg/tg*^ mice were either mock-sensitized with PBS and challenged with OVA (PBS/OVA) or sensitized and challenged with OVA (OVA/OVA) as indicated. Panel **a**, top row: H&E-stained sections of the peribronchiolar region of the lung; middle row: mMBP-positive (eosinophilic) cells; bottom row: MPO-positive (neutrophilic) cells. Panel **S**: quantification in the sections of inflammatory cells in OVA-OVA-treated *F/A2*^*wt/wt*^ (white diamonds) and *F/A2*^*tg/tg*^ mice (black diamonds). The sections that were compared were stained simultaneously. Inflammatory scores were analyzed per lung region, i.e. peribronchiolar, parenchymal and perivenous (around the lung veins), and scored on a scale from 0 to 3 by two independent observers. Medians and quartiles of 7–8 mice per group are shown (when not drawn, quartiles coincide with medians). No differences in inflammatory scores were found

We investigated whether hypoargininemia modified the allergic asthma-induced inflammatory response in lung tissue (Fig. 6). H&E-stained sections revealed no inflammatory cells in the lungs of PBS/OVA-treated mice (Fig. 6a). This finding was confirmed by staining sections of the same lungs for the presence of major basic protein (mMBP) and myeloperoxidase (MPO), eosinophil and neutrophil markers, respectively. As expected, inflammatory cells were abundant in the lungs of OVA/OVA-treated mice, but there was no difference between *F/A2*^*tg/tg*^ and *F/A2*^*wt/wt*^ mice. Semi-quantitative scoring of the sections for inflammatory cell density in the peribronchiolar, parenchymal and perivenous areas did not reveal differences between OVA/OVA-treated *F/A2*^*tg/tg*^ mice and their wild-type littermates (Fig. 6b).

### Hypoargininemia disturbs the coordinated response to asthma

**Fig. 7:**
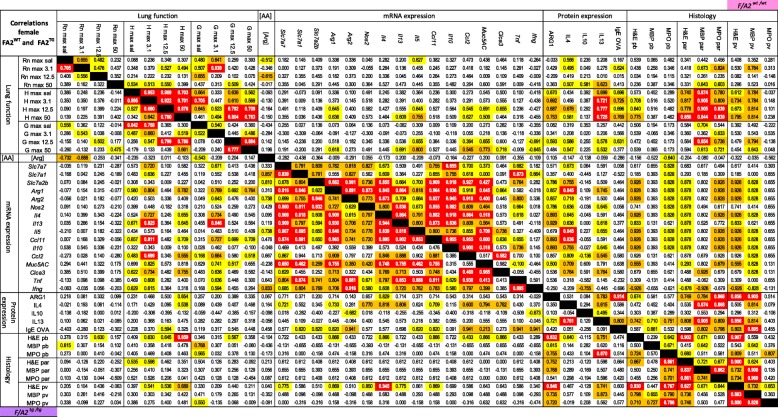
Disruption of coordinated response to allergic asthma in hypoargininemic and control mice. The correlation of parameters of lung function, plasma concentration of arginine, abundance of pulmonary mRNAs and proteins, and pulmonary histopathology were compared in control and asthmatic *F/A2*^*tg/tg*^ and wild-type littermate mice. Correlation coefficients of parameters indicated above and to the left of the columns and rows, respectively, are shown for *F/A2*^*wt/wt*^ (upper right triangle) and *F/A2*^*tg/tg*^ mice (lower left triangle). *n* = 15 or 16 PBS/OVA- and OVA/OVA-treated mice of both genotypes. The significance of the correlations is color-coded according to the *P*-value of the correlation coefficient (Spearman’s rho): yellow: 0.05 > *P* > 0.01, orange: 0.01 > *P* > 0.001, and red: *P* < 0.001

To answer the question whether circulating L-arginine concentrations changed the overall adaptive response to asthma, we determined the correlation coefficients between AHR parameters, mRNA and protein levels, histology scores and L-arginine concentrations in *F/A2*^*tg/tg*^ mice and their *F/A2*^*wt/wt*^ littermates (Fig. 7). The presence of 3 major blocs (lung function, mRNA expression, and lung histology) with high correlations between parameters in the response to OVA/OVA within a bloc and fewer significant correlations between the blocs is striking. mRNA concentrations in particular hardly correlated with function or histology. Furthermore, more significant correlations were seen in *F/A2*^*wt/wt*^ (wild-type FVB mice) than in *F/A2*^*tg/tg*^ mice, suggesting that arginine availability played a role in these responses. In more detail, we observed that significant correlations between the responses of lung function or lung histology parameters to OVA/OVA treatment were more frequently seen in *F/A2*^*wt/wt*^ than in *F/A2*^*tg/tg*^ mice. In contrast, the responses in mRNA expression to OVA/OVA treatment were similar in both types of mice, with the notable exception of the expression of *Il10* (no correlation with other mRNAs in *F/A2*^*tg/tg*^) and *Tnf* and *Ifng* (stronger correlations). Plasma arginine concentrations correlated inversely with large airway resistance. In aggregate, these data show that hypoargininemia disturbed the coordinated asthma response. The finding that tissue inflammation did not correlate well with lung function parameters in *F/A2*^*tg/tg*^ mice indicates that these events do not share a common regulatory path in this mouse model of asthma [25].

## Discussion

In this study, we investigated the symptoms of allergic asthma in hypoargininemic *F/A2*^*tg/tg*^ mice [19]. The decrease of the circulating arginine concentration as a result of arginase 1 overexpression in the enterocytes was most profound in female mice. OVA/OVA treatment did not change the plasma arginine concentration in either *F/A2*^*tg/tg*^ or *F/A2*^*wt/wt*^ mice, nor did it affect sensitization to OVA, as demonstrated by similar increases of OVA-specific IgE in both genotypes. Airway resistance and tissue elastance were markedly increased in OVA/OVA-treated *F/A2*^*tg/tg*^ mice compared to similarly treated *F/A2*^*wt/wt*^ mice, while tissue resistance in *F/A2*^*tg/tg*^ mice was more sensitive to methacholine than that of similarly treated *F/A2*^*wt/wt*^ mice. Remarkably, the increased airway resistance and tissue elastance were already evident in PBS/OVA-treated *F/A2*^*tg/tg*^ mice at high concentrations of methacholine. The abundance of arginine-transporting and -metabolizing mRNAs and that of inflammatory markers increased to an equal extent in both genotypes after OVA/OVA treatment. The only observed difference was a larger increase in mRNA abundance of *Clca3* and *Muc5ac* (trend, *p* = 0.072) in *F/A2*^*tg/tg*^ mice. *Clca3* and *Muc5ac* are involved in mucus production and expressed by lung goblet cells. The findings show that the circulating concentration of arginine has a pronounced effect of airway biomechanics, but not on inflammation.

### Overexpression of arginase 1 causes hypoargininemia

In adult mice, overexpression of arginase 1 in enterocytes makes these cells an efficient arginine sink. Compared to *F/A2*^*wt/wt*^ mice, arginine concentration in plasma of *F/A2*^*tg/tg*^ mice dropped by ~ 25% (from 126 μmol/mL to 90 μmol/mL), and ~ 50% (from 124 μmol/mL to 63 μmol/mL) in male and female *F/A2*^*tg/tg*^ mice, respectively. Please note that we reported earlier that males were more affected than females [26], but that subsequent studies, including the present (Fig. 1), revealed that this conclusion was due to mislabeled blood samples.

### Hypoargininemia does not alter the inflammatory response or the expression of arginine-metabolizing or -transporting enzymes in the lung

Elimination of *Arg1* expression in macrophages has been accomplished by bone-marrow transfer from 9 to 12-day old *Arg1*-deficient pups to congenic recipient mice [17] or by Tie2Cre-mediated deletion of floxed *Arg1* alleles in hematopoietic tissues [11, 12, 18]. Allergic airway inflammation was induced by sensitizing such mice to ovalbumin, Aspergillus, or Schistosoma eggs. Furthermore, these tests were not only carried out in T_H_1-prone C57BL/6, but also in T_H_2-prone BALB/c mice. Despite this very broad approach, these studies found no effects of *Arg1* deficiency in macrophages on allergic inflammatory responses or function tests of the lungs. The present study reports that a ~ 50% decrease in plasma arginine concentration also did not affect the expression of inflammation-associated genes or the histopathology in the lungs in response to OVA/OVA treatment, even though this low ambient arginine concentration causes a clear phenotype [19] due to the induction of the endoplasmic reticulum-stress response [27]. OVA/OVA treatment affected the expression of arginine-transporting and -metabolizing genes in *Arg1*-deficient mice [11, 12], but not in *F/A2*^*tg/tg*^ mice. This finding suggests that quantitively arginine metabolism in the lungs is largely confined to macrophages. In agreement, it was earlier reported that local arginine deficiency suppresses T-cell activation and thus limits the inflammatory response [28]. Interestingly, it was also reported that a high oral dose of L-arginine reduces airway inflammation, and T_H_2 cytokine and mucus production in a mouse model of asthma [29].

### Hypoargininemia does disrupt lung mechanics

Absence of ARG1 from macrophages did not affect the ventilatory function of allergically inflamed lungs in the studies of Niese [17] and Barron [18]. Peripheral lung function was slightly better protected in *Arg1*-deficient OVA/OVA-treated male C57BL/6 mice than in their wild-type littermates [11], but this protective effect of *Arg1* deficiency in macrophages was not found in female mice [12]. The present study shows that a ~ 50% reduction of plasma arginine concentration increases airway resistance and tissue elastance, and the sensitivity of tissue resistance to methacholine treatment in OVA/OVA-treated *F/A2*^*tg/tg*^ mice. Moreover, some of these effects were already seen in PBS/OVA-treated *F/A2*^*tg/tg*^ mice, albeit to a lesser extent. The pronounced effect of hypoargininemia on lung function is remarkable, because it apparently exerts its effect outside the macrophages. These macrophages accumulate in the peribronchiolar space of asthmatic lungs, that is, close to the smooth muscle cells of the airways (see Fig. 6) and express high levels of arginase [1]. The ambient concentration of arginine had a large effect on the contraction of perfused guinea-pig tracheal rings ex vivo [30] and a high oral dose of L-arginine reduced AHR in mice in vivo [29]. Apparently, the elimination of arginase 1 expression from the peribronchial macrophages does not result in a sufficiently high local increase in arginine concentration to reduce airway resistance or mucus production. The finding that a decrease in plasma arginine concentration does aggravate the symptoms of allergic asthma suggests that a limitation of systemic L-arginine availability impairs relaxation in airway smooth muscle cells.

One reason for the sensitivity of lung mechanics to circulating rather than local arginine concentration may be the very strong perfusion of the lung and, hence, buffering effect of plasma arginine. It seems reasonable to mechanistically ascribe the dependence of lung function on circulating arginine to arginine availability for NO production. Genetic elimination of the NOS2 enzyme has been demonstrated to aggravate AHR in OVA/OVA-treated mice and resulted in a ~ 2-fold reduction of the exhaled NO concentration [8, 10, 31]. NOS1-KO mice recruit fewer eosinophils (~ 70% of wild-type) and produce less NO (~ 10% of wild-type) in a mouse model of OVA-induced asthma, and upregulation of NOS2 is abolished [9]. NOS3-KO mice are inherently hyperresponsive to inhaled methacholine [7]. The demonstration that NOS2 produces peroxynitrite in allergic asthma [32] indicates that NOS2 already tends to produce superoxide rather than nitric oxide under this condition [16]. Superoxide production by NOS is a marker for cellular arginine or BH4 deficiency [33] and may well explain why a high oral dose of L-arginine reduced the production of markers of nitro-oxidative stress, such as nitrotyrosine [29]. In aggregate, the findings in *Nos*-deficient mice show that NO plays a prominent if not determining role in AHR and underscore our hypothesis that hypoargininemia aggravates AHR in severe allergic asthma via a decrease in NO production. These experimental findings confirm the earlier clinical observation that L-arginine availability is related to airflow obstruction in severe asthma [4].

### Limitations of the study

The experimental model we used to base our conclusion on is a transgenic model. The disadvantage of transgenic models is that the life-long exposure to in this case hypoargininemia may have additional features that could affect the response to allergic asthma. The physiologically most attractive option to assess such a potential confounder would be to supplement *F/A2*^*tg/tg*^ mice with arginine. When we carried out this experiment by treating mice twice daily for 5 days with 5 mmol/kg arginine and measuring plasma arginine concentration 6 h after the last injection [26], circulating arginine concentration in wild-type mice increased > 2-fold, but the intervention was without any effect in *F/A2*^*tg/tg*^ mice. Since circulating arginine concentrations vary directly with transgenic arginase activity in the small intestine of *F/A2*^*tg/tg*^ mice [19], the high turnover of plasma arginine in *F/A2*^*tg/tg*^ mice apparently neutralizes the supplemented arginine. An alternative option would be to infuse arginase intravenously into wild-type mice. This intervention is very effective in lowering circulating arginine concentrations, but also short-lived [34], so that it could not be used in the present setting. A third approach could have been treatment of *F/A2*^*tg/tg*^ mice with a well-established arginase inhibitor such as nor-NOHA. However, nor-NOHA is an arginine analogue which requires ~ 10-fold higher concentration to inhibit intracellular than dissolved arginase [35], suggesting it has to compete with arginine-like molecules for membrane transport. Probably more seriously, its K_i_ for mouse macrophage arginase is 50 μmol/L, that is, similar to circulating arginine concentrations in *F/A2*^*tg/tg*^ mice. These considerations show that the *F/A2*^*tg/tg*^ mouse model was instrumental in revealing a potential requirement for circulating arginine in severe allergic asthma, but that additional studies are necessary to reaffirm this function.

## Conclusion

Reduction of circulating arginine levels by ~ 50% in a mouse model of OVA-induced asthma leads to an increase in AHR as shown by the increases in R_N_, H, and G, without altering the inflammatory response or the expression of arginine-metabolizing or transporting enzymes in the lung. Ablation of arginase 1 in macrophages, on the other hand did not result in an improvement of any of the lung function parameters. We conclude that the reduction of circulating L-arginine levels rather than a macrophage-mediated increase in arginine catabolism worsens AHR.

## Additional files

Additional file 1: **Figure S1.** Arg1, Nos2, and Cd68 expression in lungs of OVA-sensitized FVB mice before (Control, n=4) and after 1 (n=12) or 6 (n=12) challenges with aerosolized OVA. (PDF 374 kb)

Additional file 2: **Tables S1.** Primer pairs used for genotyping and quantitative PCR. **Table S2.** Amino-acid concentrations in venous plasma (μM ± SEM) of *F/A2*^*wt/wt*^ and *F/A2*^*tg/tg*^ female mice. (DOCX 21 kb)

## Abbreviations

(m)MBP: (murine) major basic protein
AHR: Airway hyperresponsiveness
Arg: Arginase
CAT: Cationic amino acid transporter
Ccl11: C-C motif chemokine ligand 11
Ccl2: C-C motif chemokine ligand 2
Clca3: Calcium-activation chloride channel family member 3
F/A2: transgenic mice on a FVB background that overexpress rat arginase 1in their enterocytes
G: tissue resistance
H: tissue elastance
Ifng: interferon gamma
Il: interleukin
LAT: heterodimeric amino acid transporter
MPO: myeloperoxidase
NO: nitric oxide
NOS: nitric oxide synthetase
OVA: ovalbumin
PBS: phosphate-buffered saline
R_N_: airway resistance
RT: room temperature
Slc7a1/2/7: solute carrier family 7 members1, 2 or 7 resp.
Tnfa: tumor necrosis factor alpha; Zrs – input impedance
