# Supplementary material for: Hypoargininemia exacerbates airway hyperresponsiveness in a mouse model of asthma
Source: Respir Res. 2018 May 23;19:98. doi: 10.1186/s12931-018-0809-9 (PMC5967058; doi:10.1186/s12931-018-0809-9)
Supplement: Supplementary file 1 — Figure S1. Arg1, Nos2, and Cd68 expression in lungs of OVA-sensitized FVB mice before (Control, n=4) and after 1 (n=12) or 6 (n=12) challenges with aerosolized OVA. (PDF 374 kb) [file 12931_2018_809_MOESM1_ESM.pdf]

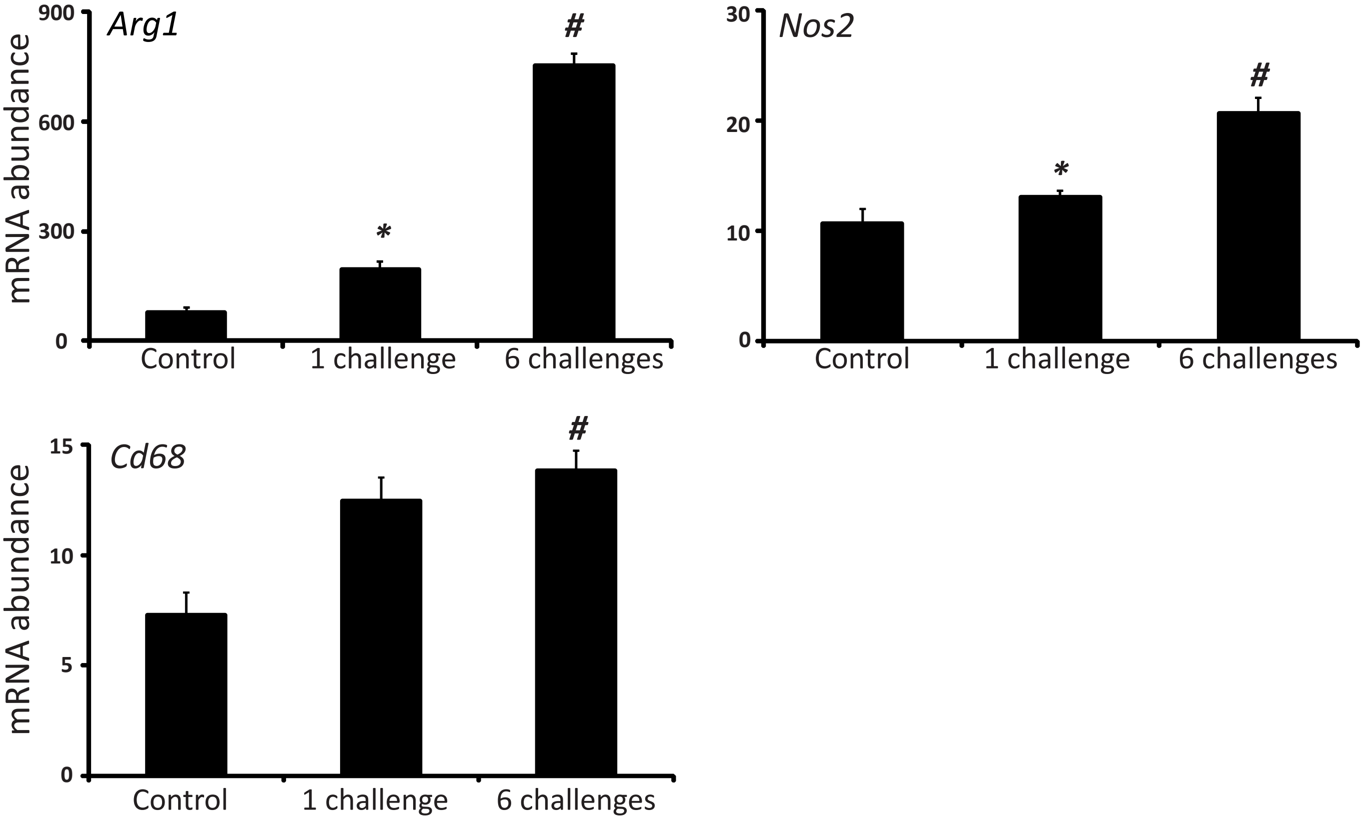

*Supplemental Figure 1S. Arg1, Nos2, and Cd68 expression in lungs of OVA-sensitized FVB mice before (Control, n=4) and after 1 (n=12) or 6 (n=12) challenges with aerosolized OVA. * = p<0.001 compared to control, # = p <0.001 compared to control*
