# Supplementary material for: Hypoargininemia exacerbates airway hyperresponsiveness in a mouse model of asthma
Source: Respir Res. 2018 May 23;19:98. doi: 10.1186/s12931-018-0809-9 (PMC5967058; doi:10.1186/s12931-018-0809-9)
Supplement: Supplementary file 2 — Tables S1. Primer pairs used for genotyping and quantitative PCR. Table S2. Amino-acid concentrations in venous plasma (μM ± SEM) of F/A2wt/wt and F/A2tg/tg female mice. (DOCX 21 kb) [file 12931_2018_809_MOESM2_ESM.docx]

**Supplemental Table 1. Primer pairs used for genotyping and quantitative PCR**

| Gene | Primer name | Primer sequence (5’-3’) |
| --- | --- | --- |
| Excised *arginase 1* | Arg1-F2 | TCTAGAACTAGTGGATCACCTCAG |
|  | Arg1-R1 | GTGCCTTGGTCTACATTGAACATAC |
| *Cre* | CRE-F | GGTTCGCAAGAACCTGATGGACAT |
|  | CRE-R | GCTAGAGCCTGTTTTGCACGTTCA |
| *Tie-2-Cre* | Tie2-F | CGCATAACCAGTGAAACAGCATTGC |
|  | Tie2-R | CCCTGTGCTCAGACAGAAATGAGA |
| *LysMCre* | LysM-F | GGT TCG CAA GAA CCT GAT GGA CAT |
|  | LysM-R | GCT AGA GCC TGT TTT GCA CGT TCA |
| *Arginase 1* | Arg1-F1 | GGAGAGCCTTCCTGCACTTT |
|  | Arg1-R1 | GTGCCTTGGTCTACATTGAACATAC |
| *Arginase 2* | Arg2-F | CCAGCTGCCATTCGAGAAG |
|  | Arg2-R | ATCATCTTGTGGGACATTAGTAAACTC |
| *Nos 2* | Nos2-F | GCCACCAACAATGGCAACA |
|  | Nos2-R | CGTACCGGATGAGCTGTGAATT |
| *Scl7a1* | Cat1-F | CTGGTGGACCTCATGTCCATT |
|  | Cat1-R | GCTCATTCTGATCTACTCGATCTAGCT |
| *Slc7a2b* | Cat2b-F | GATCCATTTTCCCAATGCCTC |
|  | Cat2b-R | TGGAATTGATTTGAGCTAGACATTTG |
| *Slc7a7* | Lat1-F | GAAGGACCCCGACCGG |
|  | Lat1-R | AACAGCCACCAGGAAGATGG |
| *Il4* | IL4-F | TGGAATGTACCAGGAGCCATATCC |
|  | IL4-R | CTCTGTGGTGTTCTTCGTTGCTGT |
| *Il13* | IL13-F | CACACAAGACCAGACTCCCCTG |
|  | IL13-R | GGTTACAGAGGCCATGCAATATCC |
| *Il5* | IL5-F | ATCAAACTGTCCGTGGGGGTACT |
|  | IL5-R | TCTCTCCTCGCCACACTTCTCTTT |
| *Il10* | IL10-F | GGACAACATACTGCTAACCGACTCCT |
|  | IL10-R | CTGCTCCACTGCCTTGCTCTTATT |
| *Ccl2* | MCP1-F | GCTGGAGAGCTACAAGAGGAT |
|  | MCP1-R | ACAGACCTCTCTCTTGAGCTTGGT |
| *Ccl11* | Eotaxin1-F | CTGCTGCTCACGGTCACTTCCT |
|  | Eotaxin1-R | CAGGGTGCATCTGTTGTTGGTG |
| *Ifng* | IFNG-F | GGTTGCTCCTCTTACCGTCTTT |
|  | INFG-R | CGTGGCACTTTTTACCACAGA |
| *Tnfa* | TNFA-F | TCAATCGGCCCGACTATCTC |
|  | TNFA-R | CAGGGCAATTGATCCCAAAGT |
| *Muc5ac* | MUC5AC-F | GATGACTTCCAGACTATCAGTG |
|  | MUC5AC-R | TGGCGTTAGTCAGCAGA |
| *Clca3* | CLCA3-F | CCGGCTGCCGCTAAAGAG |
|  | CLCA3-R | CAGAAGCATCAACAAGACCATTG |
| *18S* | 18S-F | AGTTAGCATGCCAGAGTCTCG |
|  | 18S-R | TGCATGGCCGTTCTTAGTTG |

|  | *F/A2^wt/wt^* | | *F/A2^tg/tg^* | |
| --- | --- | --- | --- | --- |
| Amino acid | PBS/OVA | OVA/OVA | PBS/OVA | OVA/OVA |
| Asp | 47 ± 23 | 25 ± 4 | 32 ± 13 | 12 ± 3 |
| Glu | 61 ± 17 | 36 ± 6 | 55 ± 16 | 41 ± 11 |
| Asn | 63 ± 17 | 40 ± 4 | 45 ± 3 | 51 ± 7 |
| Ser | 188 ± 33 | 143 ± 21 | 141 ± 24 | 128 ± 16 |
| Gln | 813 ± 70 | 730 ± 58 | 790 ± 42 | 720 ± 34 |
| His | 110 ± 9 | 94 ± 5 | 111 ± 7 | 102 ± 6 |
| Thr | 198 ± 20 | 165 ± 20 | 203 ± 14 | 214 ± 23 |
| 3-MeHIS | 17 ± 5 | 6 ± 1 | 12 ± 2 | 9 ± 3 |
| Cit | 91 ± 11 | 70 ± 7 | 70 ± 4 | 71 ± 6 |
| Arg | 130 ± 16 | 118 ± 8 | 61 ± 5 ***** | 65 ± 9 # |
| Ala | 941 ± 95 | 914 ± 70 | 1020 ± 78 | 1088 ± 98 |
| Tyr | 188 ± 34 | 143 ± 12 | 149 ± 18 | 157 ± 19 |
| Tau | 270 ± 36 | 210 ± 19 | 283 ± 19 | 250 ± 38 |
| Val | 304 ± 24 | 241 ± 14 | 280 ± 25 | 240 ± 22 |
| Met | 79 ± 6 | 61 ± 3 | 71 ± 7 | 62 ± 6 |
| Ile | 173 ± 17 | 132 ± 9 | 150 ± 11 | 130 ± 11 |
| Phe | 89 ± 12 | 74 ± 10 | 77 ± 5 | 70 ± 13 |
| Orn | 92 ± 13 | 78 ± 5 | 82 ± 7 | 80 ± 12 |
| Leu | 225 ± 25 | 198 ± 9 | 231 ± 13 | 200 ± 7 |
| Trp | 265 ± 23 | 260 ± 21 | 246 ± 5 | 246 ± 21 |
| Lys | 475 ± 47 | 473 ± 33 | 468 ± 23 | 423 ± 29 |
| total AAs | 4798 ± 442 | 4183 ± 270 | 4577 ± 202 | 4353 ± 316 |
| Arg/Orn | 1.8 ± 0.5 | 1.6 ± 0.1 | 0.8 ± 0.1 ***** | 0.9 ± 0.1 # |
| Arg/Orn+Cit) | 0.76 ± 0.11 | 0.83 ± 0.04 | 0.42 ± 0.04 * | 0.45 ± 0.04 # |
| Arg/(Orn+Lys) | 0.23 ± 0.02 | - 1. ± 0.01 | 0.11 ± 0.01 ***** | 0.11 ± 0.01 # |

**Supplemental Table 2:** Amino-acid concentrations in venous plasma (µM ± SEM) of *F/A2^wt/wt^* and *F/A2^tg/tg^* female mice. * = P< 0.05 for PBS/OVA *F/A2^tg/tg^* vs. PBS/OVA *F/A2^wt/wt^*;

# = P<0.05 for OVA/OVA *F/A2^tg/tg^* vs. OVA/OVA *F/A2^wt/wt^*.
